# 3-Chloro­propio­phenone

**DOI:** 10.1107/S2414314625003499

**Published:** 2025-04-29

**Authors:** Marcel Sonneck, Anke Spannenberg, Sebastian Wohlrab, Tim Peppel

**Affiliations:** a Leibniz-Institut für Katalyse e V Albert-Einstein-Str 29a 18059 Rostock Germany; Goethe-Universität Frankfurt, Germany

**Keywords:** crystal structure, 3-chloro­propio­phenone, β-chloro ketone

## Abstract

The title compound, 3-chloro­propio­phenone (or 3-chloro-1-phenyl­propan-1-one), C_9_H_9_ClO, consists of an almost planar mol­ecule that is charaterized by very small torsion angles within the alkyl side chain (torsion angles < 6.3°). No hydrogen bonds are observed in the crystal packing. The compound exhibits a melting point of 54°C.

## Abstract 2

The title compound, 3-chloro­propio­phenone C_9_H_9_ClO, consists of an almost planar mol­ecule that is charaterized by very small torsion angles within the alkyl side chain (torsion angles < 6.3°).

## Graphical abstract

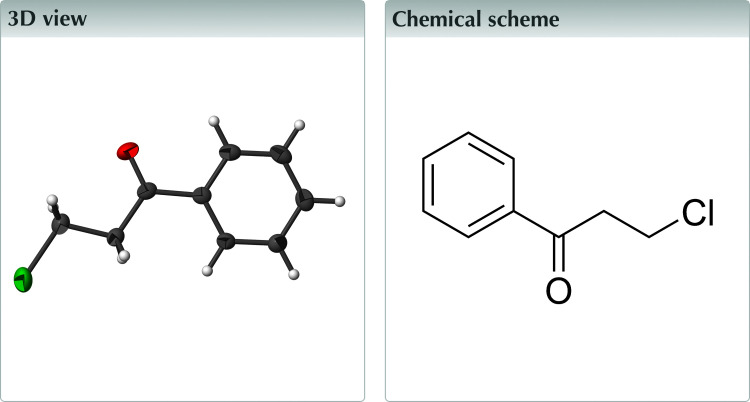

## Structure description

β-Chloro ketones are useful building blocks for many chemical transformation reactions. They are accessible *via* different reaction schemes such as Friedel-Crafts acyl­ation (Sartori & Maggi, 2006 ▸), Wacker-type oxidation (Liu *et al.*, 2017 ▸), or light-mediated ring opening of aryl cyclo­propanes (Petzold *et al.*, 2019 ▸). The title compound was obtained in almost qu­anti­tative yield in high purity. It can be designated as a suitable building block in the ongoing efforts to synthesize feasible new ligands for Cu-based complexes (Sonneck *et al.*, 2015 ▸, 2016 ▸).

**Figure 1:**
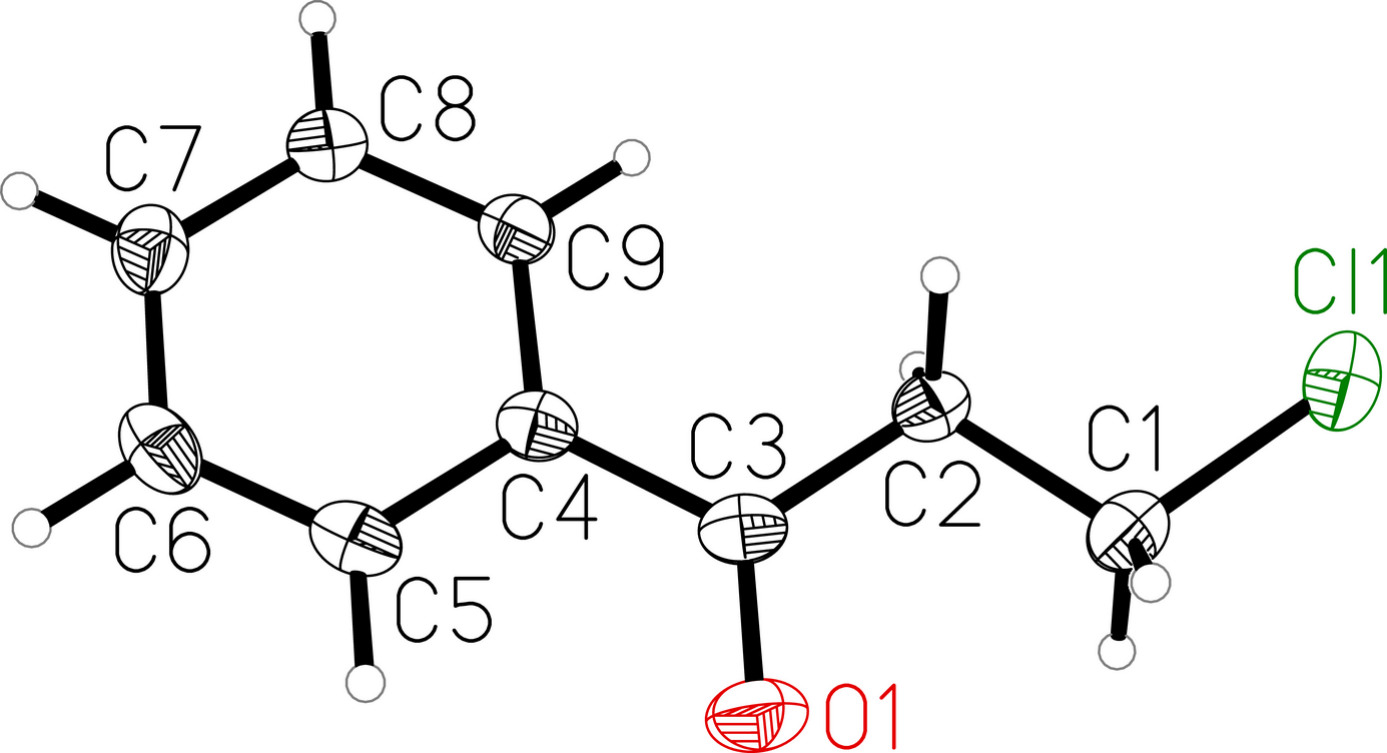
Mol­ecular structure of the title compound with atom labelling and displacement ellipsoids drawn at 50% probability level.

The mol­ecular structure of 3-chloro­propio­phenone is almost planar with torsion angles of less than 6.3 degrees [maximum torsion angle: C1—C2—C3—O1 = −6.21 (19)°] in the side chain (Fig. 1 ▸). The main deviation out of the plane defined by the non-hydrogen atoms of the mol­ecule is observed for O1 with −0.1091 (10) Å and for Cl1 with 0.1065 (8) Å. In addition, the layered packing prevents the formation of extended halogen or hydrogen-bonding networks. The mol­ecules form stacks along the *c* axis. In a stack, neighbouring mol­ecules are related by the *c* glide plane. All bond lengths and angles are within the expected range and the C=O bond is 1.2158 (18) Å.

## Synthesis and crystallization

3-Chloro­propio­phenone was obtained as colourless crystals in qu­anti­tative yield from the Friedel–Crafts acyl­ation of benzene and 3-chloro­propionyl chloride in di­chloro­methane. AlCl_3_ (38.2 g, 286.5 mmol, 1.25 eq.) was suspended in 50 ml of dry di­chloro­methane at 0°C. A solution of 3-chloro­propionyl chloride (29.1 g, 229.2 mmol, 1.0 eq.) in 90 ml di­chloro­methane was added dropwise at 0°C to the AlCl_3_ suspension. Afterwards, a solution of benzene (17.9 g, 229.2 mmol, 1.0 eq.) in 25 mL di­chloro­methane was added dropwise at 0°C to the suspension and further stirred for 2 h at 0°C and 12 h at ambient conditions. The final solution was poured onto ice and concentrated hydro­chloric acid (70 g:7 g) and after separation of the organic phase, the aqueous phase was extracted twice with 100 ml portions of di­chloro­methane. The combined organic phases were extracted twice with 150 ml portions of water and finally dried over Na_2_SO_4_. The solvent was removed completely under diminished pressure and the off-white crystalline solid residue was recrystallized from pentane to yield the final product (37.5 g, 97%). Colourless single crystals of 3-chloro­propio­phenone were obtained from a pentane solution by slow evaporation of the solvent at 4°C over the period of one week. Analytic data for C_9_H_9_ClO: m.p. 54°C, elemental analysis % (calculated): C 64.14 (64.11), H 5.25 (5.38); Cl 21.01 (21.02). ^1^H NMR (400 MHz, CDCl_3_): δ (p.p.m.) = 7.98–7.93 (*m*, 2H, ArH); 7.61–7.56 (*m*, 1H, ArH); 7.51–7.45 (*m*, 2H, ArH); 3.92 (*t*, ^3^*J* = 6.8 Hz, 2H); 3.45 (*t*, ^3^*J* = 6.7 Hz, 2H); ^13^C NMR (100 MHz, CDCl_3_): δ (p.p.m.) = 196.78 (CO); 136.45 (C); 133.65, 128.84, 128.84, 128.14, 128.14 (CH); 41.36, 38.79 (CH_2_).

## Refinement

Crystal data, data collection and structure refinement details are summarized in Table 1 ▸.

## Supplementary Material

Crystal structure: contains datablock(s) I. DOI: 10.1107/S2414314625003499/bt4169sup1.cif

Structure factors: contains datablock(s) I. DOI: 10.1107/S2414314625003499/bt4169Isup2.hkl

Supporting information file. DOI: 10.1107/S2414314625003499/bt4169Isup3.cml

CCDC reference: 1501322

Additional supporting information:  crystallographic information; 3D view; checkCIF report

## Figures and Tables

**Table 1:** Experimental details

Crystal data	
Chemical formula	C_9_H_9_ClO
*M*_r_	168.61
Crystal system, space group	Monoclinic, *P*2_1_/*c*
Temperature (K)	150
*a*, *b*, *c* (Å)	5.4485 (13), 20.347 (5), 7.4860 (17)
β (°)	102.123 (4)
*V* (Å^3^)	811.4 (3)
*Z*	4
Radiation type	Mo *K*α
μ (mm^−1^)	0.40
Crystal size (mm)	0.51 × 0.43 × 0.07

Data collection	
Diffractometer	Bruker APEXII CCD
Absorption correction	Multi-scan (*SADABS*; Krause *et al.*, 2015 ▸)
*T*_min_, *T*_max_	0.79, 0.97
No. of measured, independent and observed [*I* > 2σ(*I*)] reflections	9113, 1951, 1595
*R*_int_	0.032
(sin θ/λ)_max_ (Å^−1^)	0.661

Refinement	
*R*[*F*^2^ > 2σ(*F*^2^)], *wR*(*F*^2^), *S*	0.038, 0.095, 1.08
No. of reflections	1951
No. of parameters	100
H-atom treatment	H-atom parameters constrained
Δρ_max_, Δρ_min_ (e Å^−3^)	0.36, −0.24

Computer programs: *APEX2* (Bruker, 2014 ▸), *SAINT* (Bruker, 2013 ▸), *SHELXS97* (Sheldrick, 2008 ▸), *SHELXL2014/7* (Sheldrick, 2015 ▸), *XP* in *SHELXTL* (Sheldrick, 2008 ▸) and *publCIF* (Westrip, 2010 ▸).

## full crystallographic data

### 3-Chloro-1-phenylpropan-1-one Crystal data

**Table:**

C_9_H_9_ClO	*F*(000) = 352
*M**_r_* = 168.61	*D*_x_ = 1.380 Mg m^−^^3^
Monoclinic, *P*2_1_/*c*	Mo *K*α radiation, λ = 0.71073 Å
*a* = 5.4485 (13) Å	Cell parameters from 2965 reflections
*b* = 20.347 (5) Å	θ = 3.0–28.7°
*c* = 7.4860 (17) Å	µ = 0.40 mm^−^^1^
β = 102.123 (4)°	*T* = 150 K
*V* = 811.4 (3) Å^3^	Plate, colourless
*Z* = 4	0.51 × 0.43 × 0.07 mm

### 3-Chloro-1-phenylpropan-1-one Data collection

**Table:**

Bruker APEXII CCD diffractometer	1951 independent reflections
Radiation source: fine-focus sealed tube	1595 reflections with *I* > 2σ(*I*)
Detector resolution: 8.3333 pixels mm^-1^	*R*_int_ = 0.032
φ and ω scans	θ_max_ = 28.0°, θ_min_ = 2.0°
Absorption correction: multi-scan (SADABS; Krause *et al.*, 2015)	*h* = −7→7
*T*_min_ = 0.79, *T*_max_ = 0.97	*k* = −26→25
9113 measured reflections	*l* = −9→9

### 3-Chloro-1-phenylpropan-1-one Refinement

**Table:**

Refinement on *F*^2^	Primary atom site location: structure-invariant direct methods
Least-squares matrix: full	Hydrogen site location: inferred from neighbouring sites
*R*[*F*^2^ > 2σ(*F*^2^)] = 0.038	H-atom parameters constrained
*wR*(*F*^2^) = 0.095	*w* = 1/[σ^2^(*F*_o_^2^) + (0.0414*P*)^2^ + 0.259*P*] where *P* = (*F*_o_^2^ + 2*F*_c_^2^)/3
*S* = 1.08	(Δ/σ)_max_ < 0.001
1951 reflections	Δρ_max_ = 0.36 e Å^−^^3^
100 parameters	Δρ_min_ = −0.24 e Å^−^^3^
0 restraints	

### 3-Chloro-1-phenylpropan-1-one Special details

**Table:**

Geometry. All esds (except the esd in the dihedral angle between two l.s. planes) are estimated using the full covariance matrix. The cell esds are taken into account individually in the estimation of esds in distances, angles and torsion angles; correlations between esds in cell parameters are only used when they are defined by crystal symmetry. An approximate (isotropic) treatment of cell esds is used for estimating esds involving l.s. planes.
Refinement. Hydrogen atoms were located in a difference map and refined as riding on their parent atoms with *U*(H)=1.2*U*_eq_(C).

### 3-Chloro-1-phenylpropan-1-one Fractional atomic coordinates and isotropic or equivalent isotropic displacement parameters (Å^2^)

**Table:**

	*x*	*y*	*z*	*U*_iso_*/*U*_eq_	
C1	0.4181 (3)	0.91110 (7)	0.3732 (2)	0.0292 (3)	
H1A	0.5586	0.9205	0.3120	0.035*	
H1B	0.4812	0.9154	0.5067	0.035*	
C2	0.3239 (3)	0.84182 (7)	0.3283 (2)	0.0243 (3)	
H2A	0.1794	0.8331	0.3859	0.029*	
H2B	0.2658	0.8373	0.1944	0.029*	
C3	0.5285 (3)	0.79232 (7)	0.39585 (19)	0.0252 (3)	
C4	0.4665 (3)	0.72090 (7)	0.38053 (19)	0.0229 (3)	
C5	0.6530 (3)	0.67574 (8)	0.4561 (2)	0.0283 (3)	
H5	0.8139	0.6911	0.5163	0.034*	
C6	0.6055 (3)	0.60927 (8)	0.4438 (2)	0.0313 (4)	
H6	0.7334	0.5789	0.4953	0.038*	
C7	0.3713 (3)	0.58649 (8)	0.3564 (2)	0.0309 (3)	
H7	0.3389	0.5406	0.3484	0.037*	
C8	0.1843 (3)	0.63050 (8)	0.2808 (2)	0.0280 (3)	
H8	0.0241	0.6148	0.2205	0.034*	
C9	0.2314 (3)	0.69757 (7)	0.29323 (19)	0.0245 (3)	
H9	0.1026	0.7277	0.2419	0.029*	
Cl1	0.17021 (9)	0.96882 (2)	0.29882 (7)	0.04702 (17)	
O1	0.7414 (2)	0.81030 (6)	0.46074 (17)	0.0399 (3)	

### 3-Chloro-1-phenylpropan-1-one Atomic displacement parameters (Å^2^)

**Table:**

	*U*^11^	*U*^22^	*U*^33^	*U*^12^	*U*^13^	*U*^23^
C1	0.0317 (8)	0.0274 (7)	0.0294 (8)	−0.0056 (6)	0.0081 (6)	−0.0028 (6)
C2	0.0243 (7)	0.0254 (7)	0.0237 (7)	−0.0038 (5)	0.0064 (6)	−0.0007 (5)
C3	0.0227 (7)	0.0327 (8)	0.0202 (7)	−0.0042 (6)	0.0050 (6)	0.0002 (6)
C4	0.0221 (7)	0.0290 (7)	0.0190 (6)	−0.0001 (5)	0.0074 (6)	0.0011 (5)
C5	0.0214 (7)	0.0381 (8)	0.0254 (8)	0.0028 (6)	0.0051 (6)	0.0014 (6)
C6	0.0298 (8)	0.0356 (8)	0.0303 (8)	0.0108 (6)	0.0103 (7)	0.0052 (6)
C7	0.0375 (9)	0.0266 (7)	0.0317 (8)	0.0034 (6)	0.0144 (7)	0.0011 (6)
C8	0.0256 (8)	0.0295 (7)	0.0294 (8)	−0.0017 (6)	0.0065 (6)	−0.0001 (6)
C9	0.0216 (7)	0.0277 (7)	0.0243 (7)	0.0015 (5)	0.0052 (6)	0.0027 (5)
Cl1	0.0454 (3)	0.0260 (2)	0.0684 (3)	0.00011 (18)	0.0092 (2)	−0.00376 (19)
O1	0.0255 (6)	0.0408 (7)	0.0487 (8)	−0.0080 (5)	−0.0034 (5)	0.0026 (5)

### 3-Chloro-1-phenylpropan-1-one Geometric parameters (Å, º)

**Table:**

C1—C2	1.514 (2)	C4—C5	1.399 (2)
C1—Cl1	1.7882 (17)	C5—C6	1.377 (2)
C1—H1A	0.9900	C5—H5	0.9500
C1—H1B	0.9900	C6—C7	1.386 (2)
C2—C3	1.509 (2)	C6—H6	0.9500
C2—H2A	0.9900	C7—C8	1.385 (2)
C2—H2B	0.9900	C7—H7	0.9500
C3—O1	1.2158 (18)	C8—C9	1.388 (2)
C3—C4	1.491 (2)	C8—H8	0.9500
C4—C9	1.393 (2)	C9—H9	0.9500
			
C2—C1—Cl1	110.12 (11)	C5—C4—C3	118.39 (13)
C2—C1—H1A	109.6	C6—C5—C4	120.52 (14)
Cl1—C1—H1A	109.6	C6—C5—H5	119.7
C2—C1—H1B	109.6	C4—C5—H5	119.7
Cl1—C1—H1B	109.6	C5—C6—C7	120.13 (14)
H1A—C1—H1B	108.2	C5—C6—H6	119.9
C3—C2—C1	110.81 (12)	C7—C6—H6	119.9
C3—C2—H2A	109.5	C8—C7—C6	120.13 (15)
C1—C2—H2A	109.5	C8—C7—H7	119.9
C3—C2—H2B	109.5	C6—C7—H7	119.9
C1—C2—H2B	109.5	C7—C8—C9	119.92 (15)
H2A—C2—H2B	108.1	C7—C8—H8	120.0
O1—C3—C4	120.39 (14)	C9—C8—H8	120.0
O1—C3—C2	120.58 (14)	C8—C9—C4	120.33 (14)
C4—C3—C2	119.03 (12)	C8—C9—H9	119.8
C9—C4—C5	118.96 (14)	C4—C9—H9	119.8
C9—C4—C3	122.65 (13)		
			
Cl1—C1—C2—C3	−178.09 (10)	C3—C4—C5—C6	−179.35 (13)
C1—C2—C3—O1	−6.21 (19)	C4—C5—C6—C7	−0.1 (2)
C1—C2—C3—C4	174.28 (12)	C5—C6—C7—C8	0.1 (2)
O1—C3—C4—C9	−174.35 (14)	C6—C7—C8—C9	−0.3 (2)
C2—C3—C4—C9	5.2 (2)	C7—C8—C9—C4	0.4 (2)
O1—C3—C4—C5	5.2 (2)	C5—C4—C9—C8	−0.4 (2)
C2—C3—C4—C5	−175.26 (12)	C3—C4—C9—C8	179.21 (13)
C9—C4—C5—C6	0.2 (2)		
